# The future of multimorbidity management in the older adults: transforming AI-enabled precision medicine

**DOI:** 10.3389/fpubh.2025.1691682

**Published:** 2026-01-12

**Authors:** Wei Deng, Li-Ying Zhang, Ji-Rong Yue, Xiao-Li Huang

**Affiliations:** The Center of Gerontology and Geriatrics, National Clinical Research Center for Geriatrics, West China Hospital, Sichuan University, Chengdu, Sichuan, China

**Keywords:** artificial intelligence, geriatric care, multimorbidity, personalized intervention, precision medicine

## Abstract

With global population aging, the prevalence of multimorbidity among older adults has risen sharply. This growing complexity challenges traditional single-disease-oriented healthcare models, leading to fragmented care, increased polypharmacy risks, and poor clinical outcomes. Precision medicine, integrating genomic, phenotypic, and behavioral data, offers a promising avenue for individualized care in this context. Concurrently, artificial intelligence (AI) has emerged as a powerful enabler of precision medicine by facilitating large-scale data analysis, real-time risk prediction, and multimodal data integration. This review summarizes recent advances in the application of AI-enabled precision medicine for managing geriatric multimorbidity, providing a theoretical and practical framework for integrating AI-enabled care. It highlights the need for interdisciplinary collaboration, regulatory innovation, and equity-focused design to transform multimorbidity management in aging societies.

## 1 Introduction

As the global population ages, multimorbidity has become a complex and urgent health challenge. Multimorbidity, commonly defined as the coexistence of two or more chronic conditions, significantly impacts quality of life, treatment burden, and healthcare costs in older adults (1–3). While precision medicine has offered personalized approaches by leveraging genetic, clinical, and environmental data (4), the complexity of multimorbidity often exceeds the capabilities of traditional disease-centered strategies.

**Figure 1:**
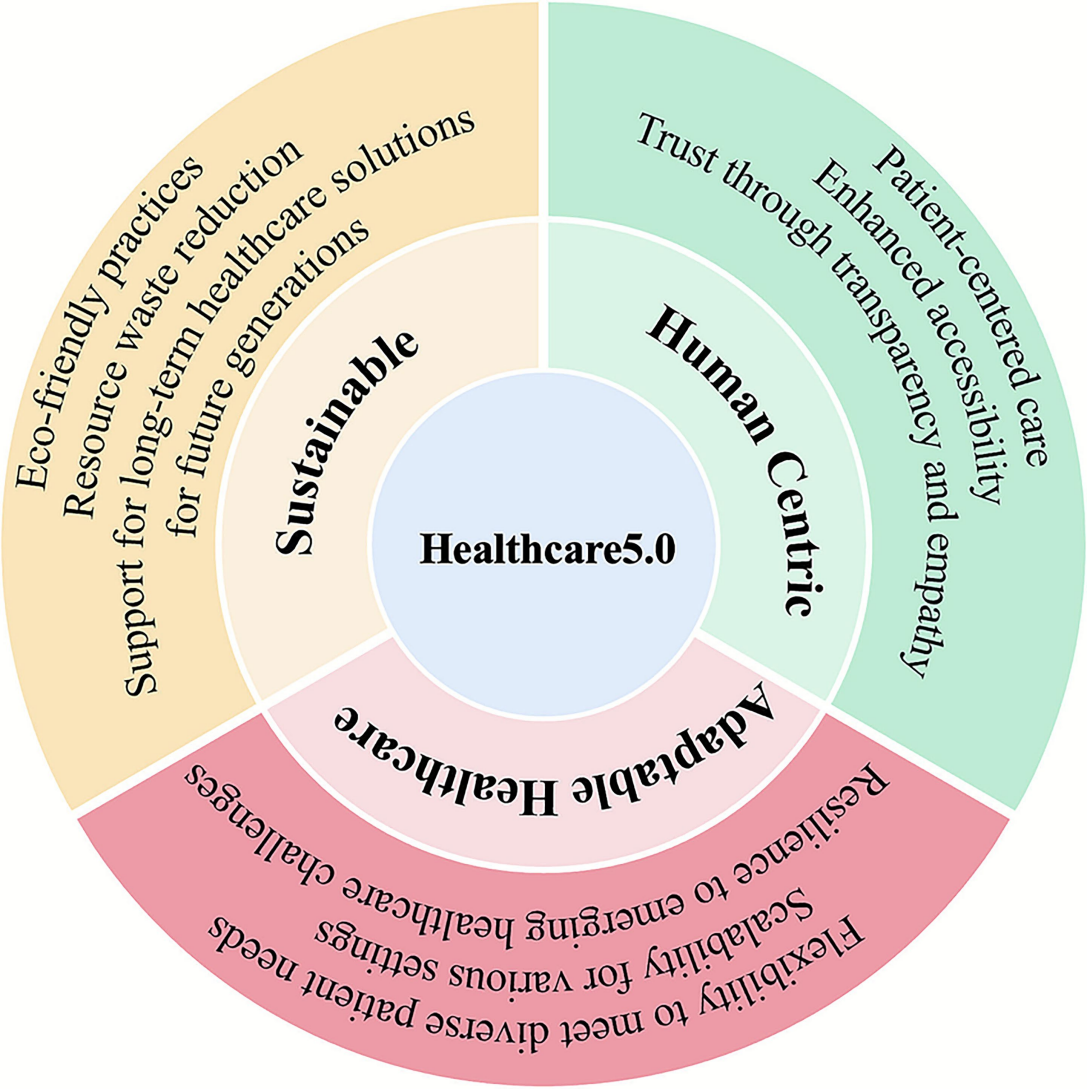
Healthcare 5.0 framework. The framework is built on three core pillars: human-centric care (emphasizing patient-centered approaches, transparency, and empathy), adaptable healthcare (ensuring flexibility, scalability, and resilience), and sustainable (promoting eco-friendly practices, resource efficiency, and long-term solutions). This framework aims to achieve improved, sustainable, accessible, and affordable healthcare outcomes.

To address this complexity, the emerging paradigm of Healthcare 5.0 has gained attention as a holistic, human-centric approach that integrates advanced digital technologies—including AI, IoT, robotics, and 6G—to provide intelligent, coordinated, and sustainable care solutions (Figure 1) (5–7). This framework extends the vision of Industry 5.0 into the healthcare domain, shifting focus from purely efficiency-driven care toward systems that are resilient, ethical, and socially inclusive (8).

Despite recent advances, existing reviews on AI-enabled precision medicine tend to focus narrowly on single-technology use cases or predictive models, often ignoring the broader system-level needs of multimorbid older patients, such as cross-domain care coordination, clinician-AI collaboration, and long-term labor sustainability (9, 10). In addition, the lack of structured frameworks to categorize AI systems by their level of autonomy limits the practical deployment of decision-support tools in complex, real-world settings (11).

To fill these gaps, this review proposes a novel autonomy-based framework within the extended Healthcare 5.0 architecture to systematically map the roles of AI in multimorbidity management. The framework classifies AI applications across four autonomy tiers—from clinician-guided tools to adaptive, agentic systems—highlighting how each can support intervention optimization, patient stratification, and care personalization in older populations.

Furthermore, this review integrates labor-sustainability, fairness, and explainability as critical axes of ethical deployment, echoing calls for AI systems that are not only effective but also just and transparent in their application to vulnerable populations (12, 13). Through this multi-dimensional perspective, we aim to redefine how AI-enabled precision medicine can support sustainable and equitable care for older patients with multimorbidity, moving beyond what AI can do to what AI should do.

## 2 Key technologies of AI-enabled precision medicine

AI-enabled precision medicine has opened new avenues for managing multimorbidity in older populations. We outline four key AI technologies—machine learning (ML), natural language processing (NLP), bioinformatics and big data analytics, and computer vision (CV)—that are increasingly contributing to individualized, data-driven care (14).

### 2.1 ML and deep learning (DL) in multimorbidity management

The ML and DL techniques have shown promise in predicting and managing comorbidities and multimorbidity. For example, one study employed various ML models—including extreme gradient boosting and convolutional neural networks (CNNs)—to achieve high accuracy in predicting chronic disease comorbidities (15). A systematic review identified 61 ML models for comorbidity prediction, many achieving high accuracy and AUC scores (16). These methods can process large medical datasets [images, charts, electronic health records (EHRs)] to effectively predict, diagnose, and guide treatment of diseases (17). Advanced ML techniques (e.g., matrix decomposition, DL, and topological data analysis) can reveal evolving patterns of multimorbidity and potential causal relationships among diseases (18). However, challenges remain in standardizing assessment methods for interpretable AI and in expanding studies to a broader range of comorbid conditions (16). Although ML offers strong predictive power, it lacks the contextual depth captured by NLP from unstructured data.

### 2.2 NLP for unstructured health data

The NLP plays a vital role in parsing clinical data by extracting and structuring information from unstructured medical text (19). NLP systems can convert complex narrative data from EHRs into structured formats, improving data accuracy and enabling better utilization by clinical information systems (20). These systems are essential for unlocking clinically important information from clinical notes to support decision-making, quality assurance, and public health initiatives (21). Thus, NLP serves as a critical complement to structured ML models, bridging the gap between quantitative metrics and qualitative patient narratives (22).

### 2.3 Bioinformatics and big data analytics in precision care

Bioinformatics and big data analytics are crucial role for advancing precision medicine. Integration of multi-organism data and EHRs offers an unprecedented opportunity for personalized healthcare (23). However, the exponential growth of biomedical big data poses significant challenges for data management, analysis, and interpretation (24). Key application areas include disease biomarker identification, patient subtyping and drug repurposing (25). Advanced computational methods are needed to address issues like data heterogeneity, missing values, and scalability (24). Big data analytics can extract valuable insights from complex datasets, improve healthcare outcomes, and enable a paradigm shift toward precision medicine (23). Nonetheless, challenges remain in data integration and interpretation, and new tools and methods must be developed to fully exploit big data’s potential in precision medicine (24, 25).

### 2.4 CV and medical image analysis

Medical imaging data has become increasingly important in managing multimorbidity among the older adults (26, 27), especially as aging and multiple chronic conditions complicate image interpretation (27). CV techniques diagnostic accuracy and efficiency through automated image processing and analysis, aiding early disease detection, disease course monitoring, and overall management of multimorbidity in the older populations (28).

Various imaging modalities [computed tomography (CT), magnetic resonance imaging (MRI), ultrasound, etc.] are essential tools for the diagnosing of multimorbidity in the older adults (29). However, the complexity and volume of image data makes manual interpretation challenging in terms of accuracy and efficiency. DL, particularly CNNs, has made significant progress in medical image analysis (30). For example, CNNs have been used to identify cerebrovascular lesions, brain atrophy, and other age-related features in CT and MRI images, enabling earlier detection of Alzheimer’s disease (31). Similarly, CV techniques have been applied in chest CT images for early lung cancer detection, helping to pinpoint tumors and improve early intervention chances (32). CV can also track changes in tumor morphology on serial imaging to assess treatment response and disease progression. In cardiovascular care, CV is widely used to analyze cardiac MRI and ultrasound for early detection of myocardial infarction and heart failure, and other conditions. For instance, regular analysis of cardiac MRI with CV can monitor structural and functional changes over time, indicating whether heart disease is worsening (33). In ultrasound imaging, CV methods have been used to detect features of cardiovascular and liver disease, reducing human error and increasing detection efficiency through automation (34, 35).

### 2.5 Comparative analysis and synergy of AI

Although individual AI modalities demonstrate clear strengths, none can independently meet the complexity of multimorbidity in older adults.

**Figure 2:**
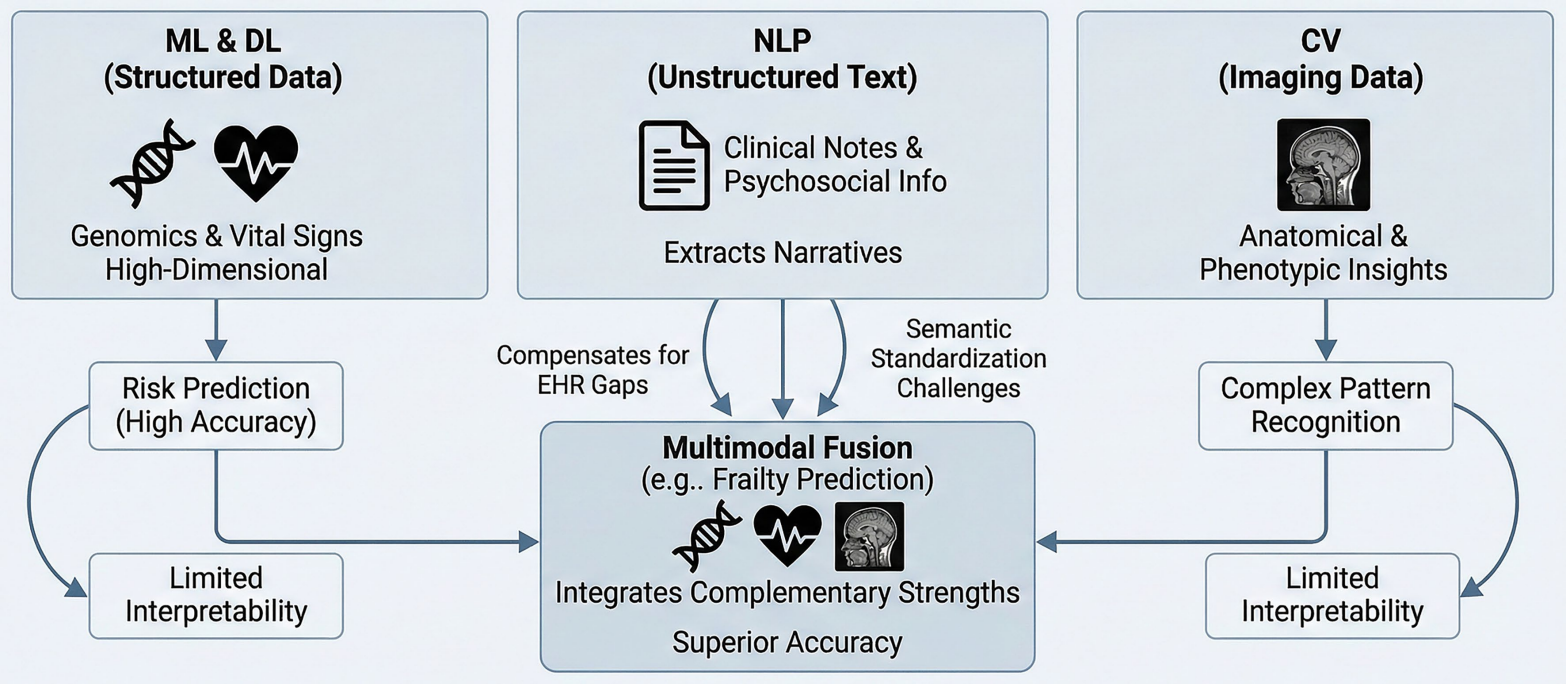
Complementary roles of AI modalities in healthcare. This figure illustrates how distinct AI methods leverage unique data sources: ML and DL excel in risk prediction using structured data, NLP extracts insights from unstructured narratives to fill EHR gaps, and CV provides anatomical insights from imaging. Multimodal fusion integrates these complementary strengths to overcome individual limitations and achieve superior accuracy in complex assessments.

The ML and DL are highly effective for structured, high-dimensional data such as genomics and vital signs, particularly in risk prediction, yet it often fails to capture the nuanced information contained in clinical narratives. NLP compensates for gaps in EHRs by extracting unstructured text such as clinical notes and psychosocial information, although it is limited by challenges in semantic standardization (36). CV provides anatomical and phenotypic insights that other modalities cannot replace. DL models achieve superior performance in complex pattern recognition, especially imaging-based tasks, but often lack interpretability. In contrast, traditional machine learning approaches offer greater transparency, albeit with slightly lower predictive capabilities. Because these methods exhibit complementary strengths and limitations, future development should prioritize multimodal fusion. For example, integrating NLP-derived social determinants with CV-based imaging biomarkers yields more accurate frailty prediction than any single modality alone (Figure 2) (37).

## 3 Clinical applications in geriatric multimorbidity

### 3.1 Autonomy-based AI framework in multimorbidity management

To clarify the role of AI in managing multimorbidity in older adults, this paper adopts a three-tier framework based on functional autonomy, distinguishing AI systems not by technical type alone but by increasing levels of autonomy, each addressing a core challenge in multimorbidity care.

Level 1: Perceptive and Descriptive AI focuses on digitizing and integrating fragmented health data. Using NLP, computer vision, and IoT sensing, it generates multimodal patient profiles that support clinical decision-making, with typical applications in remote monitoring and data fusion (38).

Level 2: Predictive and Analytical AI builds on this foundation by identifying complex patterns and forecasting disease trajectories, addressing clinical complexity. Through machine learning risk scoring and deep learning prognostic models, it enables risk stratification, early intervention, and prediction of disease progression (39).

Level 3: Prescriptive and agentic AI represents the most advanced stage, shifting from prediction to autonomous optimization and closed-loop care management. Using reinforcement learning, agent-based systems, and generative AI, it not only generates treatment recommendations but also coordinates multidisciplinary teams, dynamically adjusts therapy (e.g., medication dosing), and optimizes resource allocation. This level serves as the operational engine for achieving the adaptive and sustainable goals of Healthcare 5.0 (40).

The following sections apply this framework to analyze the clinical development and application of each level.

### 3.2 Risk prediction and early intervention

AI-enabled precision medicine, representing predictive AI, has been widely applied. As the population ages, older adults often suffer from multiple chronic diseases simultaneously, posing great challenges to the health management (41). AI-enabled precision medicine has been widely applied to risk prediction in multimorbidity, and AI-based models have demonstrated strong potential for predicting various multiple chronic conditions (42, 43). These models analyze large amounts of patient data—including medical history, clinical characteristics, and imaging—to provide more accurate disease risk predictions (44). Several technologies now offer real-time monitoring of vital signs and integrate lifestyle and environmental factors via wearables and AI-assisted telecare platforms (45). By combining diverse data sources (genetic information, lifestyle habits, diet), agentic AI can provide personalized interventions, adjust treatment plans, and generate customized exercise and dietary regimens for older patients (45, 46). AI-powered personalized medicine can improve effectiveness and increase patient adherence compared to traditional approaches (45).

These emerging clinical applications represent a shift from traditional “decision-support” AI toward a more advanced paradigm of “agentic AI.” Unlike conventional systems, agentic AI is characterized by autonomous learning, reasoning, and adaptation, making it a crucial technological foundation for realizing the Healthcare 5.0 framework outlined in the introduction. In practice, agentic AI enables the adaptability of Healthcare 5.0 through autonomous coordination, such as optimizing multidisciplinary team (MDT) workflows and resolving complex polypharmacy conflicts. Likewise, it supports the sustainability of Healthcare 5.0 through autonomous optimization, including reducing operational burdens, allocating resources more efficiently, and maintaining system performance amid workforce shortages. Soon, agentic AI will make this process even more proactive: such systems will not only predict risks but autonomously recalibrate risk thresholds based on evolving patient data. Upon identifying high-risk scenarios, they will independently initiate preventive care workflows or alert multidisciplinary teams without awaiting human instruction (47).

In routine geriatric care, AI enhances fall prevention by replacing periodic manual scoring (e.g., Morse Fall Scale) with continuous analysis of gait data from wearables and environmental sensors. When a high-risk pattern is detected, the system automatically alerts nursing staff to initiate bedside precautions and generates a referral for physical therapy, turning predictive insight into actionable workflow (48).

Similarly, in frailty assessment, AI streamlines the traditionally labor-intensive Comprehensive Geriatric Assessment (CGA). By pre-populating functional status from daily activity logs, it reduces clinicians’ data-collection burden and allows geriatricians to focus on complex decision-making and individualized care planning rather than routine administrative tasks (49).

### 3.3 Personalized treatment plan generation

For older patients, creating personalized treatment plans are particularly important due to the coexistence of multiple chronic diseases. Approaches that integrate genetic analysis, pharmacogenomics, and comprehensive patient data can optimize treatment outcomes while minimize adverse effects (50, 51). For example, in anticoagulation therapy for atrial fibrillation, assessment of molecular targets, drug interactions, and genetic polymorphisms can improve safety and efficacy (52). Alzheimer’s disease management further exemplifies the complexity of treating older adults with multiple chronic conditions; it requires personalized treatment regimens that address concomitant diseases and reduce adverse drug reactions (51). Agentic AI will enable deeper personalization in the future. For instance, in multi-medication management, AI systems could continuously learn from patients’ treatment responses and side effect data, autonomously adjusting and recommending drug dosages to ensure that treatment regimens remain dynamically optimized within the complexities of coexisting conditions (47). AI streamlines polypharmacy management by supporting medication reconciliation and deprescribing (53). For older adults using 10+ medications, it acts as a “digital pharmacist,” not only flagging high-risk interactions but also identifying drugs with poor risk–benefit profiles (e.g., anticholinergics in dementia) and simulating the effects of withdrawal (54). This turns complex medication review into a prioritized, actionable decision-support process. This would enable a more proactive, patient-centered approach to care (55).

In diabetes management, agentic AI can use data from smart devices and wearables to optimize insulin delivery and adjust treatment algorithms (56). This represents the evolution to prescriptive and agentic AI, where the system autonomously adjusts. AI also enhances diagnosis, treatment planning, and drug discovery by analyzing genomic data and predicting treatment response. However, although AI-enabled personalized medicine shows great promise in improving patient outcomes and healthcare efficiency, challenges such as data privacy, ethical issues, and the need for strong regulatory frameworks must be addressed for safe and effective implementation (55).

### 3.4 Doctor-patient communication and self-management support

The complexity of multimorbidity in the older adults places higher demands on doctor-patient communication and self-management.

AI-assisted telecare platforms can significantly support self-management for older patients with chronic conditions and comorbidities (57, 58). These platforms integrate AI analysis of physiological data to provide personalized health advice, medication reminders, and automated health reports (59, 60). Smart home technologies, including environmental sensors and external memory aids, can detect vital signs, manage medications, and monitor activities of daily living (59). The integration of AI, blockchain, and wearable technologies offers a patient-centered approach to chronic disease management, ensuring data privacy and reliability (60). AI-assisted telecare also enhances patients’ self-management awareness and helps healthcare providers quickly identify potential risks (57, 58). In cognitive care, AI-enabled communication platforms provide continuous screening by analyzing linguistic features during routine telehealth conversations (e.g., reduced vocabulary, altered speech pauses). By detecting early signs of cognitive decline or delirium progression, the system triggers an EHR alert and prompts clinicians to schedule a formal assessment (e.g., MMSE or MoCA), enabling timely evaluation and intervention (61). This telehealth self-management model reduces the need for older patients to visit hospitals, improves medical resource utilization efficiency, and helps patients achieve a higher quality of life (57, 59).

### 3.5 Healthcare resource optimization and decision support

Critically ill patients with multimorbidity often require prolonged hospitalization and constant supervision, and shortages of beds and nursing staff exacerbate the strain on the healthcare system. In geriatric multimorbidity management, prioritization and risk assessment are major challenges, especially when multiple comorbidities must be considered (62, 63). AI technologies can optimize hospital operations and bed allocation by predicting a patient’s condition progression, length of stay, and discharge timing (64). As a prescriptive AI coordinator, agentic AI learns from operational data to further enhance resource optimization. Beyond predicting hospitalization duration, it can autonomously learn from real-time information such as patient flow and staffing levels, dynamically adjusting allocation strategies to balance changing patient demand with organizational constraints (47). AI-driven decision support systems (DSS) can integrate patients’ clinical, imaging and longitudinal data to provide physicians with dynamic risk assessments and recommendations. These systems help physicians identify and prioritize the most urgent medical needs based on patients’ multimorbidity patterns (63).

By optimizing resource allocation and supporting clinicians, AI in geriatric multimorbidity management can increase healthcare efficiency and improve patient outcomes (65). For example, AI-based systems show promise in risk assessment, treatment planning, and improving patient adherence, their tireless operation is particularly useful for tasks such as image recognition, thereby reducing clinician workload. However, limitations such as data privacy concerns and potential legal implications must be considered (66). In geriatric care, AI also enables advanced clinical decision support systems, robotics, and remote monitoring technologies (67). While AI has the potential to improve resource utilization and patient prognosis, it should be regarded as a supportive tool to augment healthcare professionals, not as a replacement (66).

## 4 Challenges and ethical considerations

AI-enabled precision medicine offers great potential in geriatric care, but also poses significant ethical challenges. For example, maintaining the quality of the doctor-patient relationship and preserving human compassion in the era of AI is difficult (68).

Powerful AI applications, such as ML and NL, incur cost: their technical designs and data dependencies introduce a complex array of ethical, societal, and practical challenges. The effectiveness of AI systems heavily dependent on the data they utilize, yet this data is often incomplete, non-standardized, and difficult to obtain. The complexity of AI models, such as DL “black boxes,” can yield high accuracy while sacrificing transparency. Ageism in AI development may lead to stereotyping and bias, undermining fairness and inclusiveness for older people in digital societies (69–72).

These challenges are not peripheral issues; they are inherent limitations of AI technology itself.

### 4.1 Data privacy and security

In AI-enabled precision medicine, the privacy and security of personal health data have become central concerns (73). AI systems often require large volumes of sensitive personal data, including medical history, genomic data, and real-time monitoring, to provide personalized care for older adults (74, 75). These data face serious privacy and security risks during sharing and analysis.

Data de-identification is commonly used to protect patient privacy, but its effectiveness remains debated. While anonymization techniques can help protect privacy (76), they cannot completely eliminate re-identification risk (77). A systematic evaluation found that, on average, 34% of health records could be re-identified in various attacks, although this rate was significant lower when using established de-identification criteria (77). Various anonymization methods exist for different types of health data, but the risk of re-identification attacks remains, especially when multiple data sources are combined.

Encryption is a vital tool for protecting privacy. End-to-end encryption (E2EE) is widely used to secure data during transmission, and homomorphic encryption is increasingly applied in healthcare data processing, allowing computations on encrypted data without decryption (78, 79), thereby enabling analysis without compromising patient privacy (78). Blockchain combined with AI provides an additional layer of privacy protection for healthcare data (79). Wireless healthcare systems also utilize various encryption schemes to ensure secure data transmission and privacy protection, including homomorphic encryption matrix-based schemes (80).

Differential privacy has emerged as a promising technique for protecting individual privacy in healthcare data analysis and sharing. This approach adds controlled noise to data or query results, making it difficult to identify specific individuals (81). Recent work has highlighted its application in genomics, neuroimaging, and wearable device data (82, 83). Researchers have developed differential privacy algorithms for data publishing, predictive modeling, and aggregated analysis across data types (82). While differential privacy provides strong privacy guarantees, challenges remain in balancing privacy with utility, especially for small or correlated datasets (83). The field of differential privacy in health research is still in its early stages, with limited practical implementation. Further case studies and algorithms development are needed to assess privacy-utility trade-offs and promote widespread adoption in healthcare (83).

Federated learning (FL) is another emerging technique that enables secure model training across multiple organizations without pooling data to a central server (84). In FL, each institution trains models locally on its own data and shares only model parameters, which protects patient privacy (84, 85). However, traditional FL frameworks face challenges such as single points of failure and the potential for malicious participants. To overcome these issues, blockchain-assisted decentralized FL frameworks have been proposed, integrating training and mining tasks for greater security. Researchers have explored using DDS-based (decentralized data storage) FL frameworks to ensure secure transmission of model parameters via authentication, access control, and encryption (86).

### 4.2 Transparency and interpretability of models

AI and ML have shown great potential in healthcare, especially for analyzing complex data and predictive modeling (87). However, the lack of interpretability of many AI models, especially DL “black boxes,” poses a challenge for clinical adoption (87) which hinders trust and adoption by healthcare professionals and patients (88, 89), especially in multimorbidity management scenarios (90).

Explainable AI (XAI) has emerged as a solution to improve transparency, explainability, and trust in AI systems for healthcare professionals and patients (91). Implementing XAI in clinical settings is critical to address regulatory and ethical concerns. By improving clear explanations, XAI allows stakeholders (clinicians, patients, regulators) to understand the reasoning behind AI-driven recommendations, which is essential for building trust and accountability (87, 91). Explainability must be central to AI design, it is not merely a technical requirement but an ethical imperative. Its purpose extends beyond enabling clinicians to comprehend AI logic; it is fundamental to establishing trust among a broader stakeholder base encompassing patients, ethicists and regulators. In geriatric multimorbidity management, AI decision-making may involve complex ethical trade-offs, so transparent and explainable outputs are crucial to help non-technical personnel evaluate decisions, identify potential biases, and ensure accountability (92).

Researchers are developing various interpretability methods, including inherently interpretable models and feature-attribution methods (89, 93), such as Local Interpretable Model-agnostic Explanations (LIME) and SHapley Additive exPlanations (SHAP), which help identify important features in the decision-making process (94). These techniques enable users to understand model outputs and improve the reliability of AI-driven decisions.

However, in high-risk settings like older adults’ care, reliance on *post hoc* tools such as LIME or SHAP is insufficient and may even be misleading (95). These methods mainly provide local explanation for individual predictions but fall short of delivering the global transparency required by clinicians and regulators (96). Moreover, many XAI techniques—especially LIME—are only local approximations of complex models, and their explanations can be highly unstable: even clinically insignificant perturbations to input data may yield dramatically different interpretations (97). For example, an older patient receiving inconsistent explanations for similar assessments would find interpretability unreliable (95, 97). Therefore, in high-stakes clinical contexts, we should move beyond superficial post-hoc tools and instead pursue inherently interpretable models or develop new, domain-specific interpretability techniques that better capture the causal and correlational complexity of healthcare data (98, 99).

Challenges remain in developing more accurate and interpretable models and ensuring the responsible, ethical use of XAI (90, 93). Current research focuses on improving evaluation metrics, open-source tools, and datasets to increase the trustworthiness of AI systems (94). However, sustained efforts are needed to create robust interpretability standards and to integrate ethical safeguards throughout AI development.

### 4.3 Algorithmic bias and fairness

AI-enabled precision medicine has shown great potential for improving care of older patients with multiple chronic diseases. However, algorithmic bias is a major concern, as it may negatively impact fairness in geriatric comorbidity management and exacerbate health inequalities, particularly among diverse patient populations (100, 101). Biases in AI can lead to unequal diagnosis, treatment, and healthcare costs across racial, gender, and socioeconomic groups. Sources of bias include skewed data collection, genetic variation, and label variability (101). Viewing “fairness” solely through single subgroup comparisons (e.g., by race or gender) is a dangerous reductionism. To achieve fairness, AI systems must address intersectionality, recognizing that overlapping factors [e.g., socioeconomic status (SES), ethnicity, gender] collectively impact health outcomes (102). Researchers recommend human-centered AI principles and engagement with diverse stakeholders throughout the AI lifecycle to mitigate bias (101).

Empirical studies have highlighted these issues, that AI-based multimorbidity risk prediction models have been shown to underestimate cardiovascular disease risk in black patients compared with white patients (103). Lower SES is associated with poorer AI model performance and gaps in EHR data, affecting risk prediction and intervention timeliness (104). Many clinical AI datasets and studies originate from high-income countries (HICs), particularly the United States and China, resulting in poor representation of global diversity (105). This data inequality may lead to poor model performance in underrepresented populations, and may exacerbate health risks in non-Western or low-income groups (106). Overreliance on “race” as a proxy for biology is inherently problematic, since race is a social construct lacking a consistent biological foundation. Arbitrarily segmenting older patient cohorts into granular subgroups might improve model accuracy in theory, but it risks data scarcity and reinforcing harmful stereotypes. Consequently, developing “intersectional fairness” metrics and ensuring AI models account for overlapping identities are core imperatives for equity in older adults care (102).

On a global scale, equitable deployment of AI models faces challenges in validation and generalization. Extending models developed in HICs to low- and middle-income countries (LMICs) presents two major obstacles: first, LMICs often lack standardized, high-quality digital health data, making rigorous local validation nearly impossible (107); second, models trained on HIC populations implicitly learn population—specific features (genetic, disease patterns, healthcare resource disparities) that are mismatched with LMIC realities (108, 109). This validation gap is a root cause impeding equitable AI adoption. Models lacking local validation not only fail to gain clinical trust but also risk amplifying global health inequalities (2).

Looking ahead, fairness and inclusiveness of AI in multimorbidity management for the older adults must be improved on multiple fronts. Research has underscored the importance of addressing algorithmic biases in healthcare AI, as such biases can lead to inequalities in diagnosis, treatment, and billing (101). To mitigate these biases, experts recommend enhancing datasets diversity, improving algorithms, and continuously monitoring for bias impacts, especially in clinical applications (110). Strategies should focus on diverse data representation, algorithmic auditing, and embedding ethical considerations such as transparency and interpretability from the outset (110). Human-centered design principles—ensuring that AI serves patients’ needs—are recommended to address bias throughout the AI lifecycle (110). In addition, developing relevant policies and regulations can help to standardize fair AI applications in medicine, potentially reducing health inequalities caused by algorithmic bias (110).

### 4.4 Balancing technology and ethics

Balancing technological innovation with ethical considerations is essential to ensure the well-being and dignity of older patients. Relying solely on technical optimization is perilous; without proactively embedding an ethics framework centered on fairness, AI can amplify systemic inequalities. “Fair” choices made unilaterally by engineers and quantitative scientists may inadvertently encode their own biases into algorithms, exacerbating rather than alleviating healthcare disparities. Research has shown that both novice and experienced physicians are prone to diagnostic errors when following incorrect AI advice (111)—a phenomenon known as automation bias. Such automation bias can lead to medical errors and jeopardize patient safety (112).

Identifying biases in training data is only a superficial fix. Correcting bias is far from a simple technical task, as many biases stem from simplifying complex social constructs—such as “race”—into algorithm-friendly metrics. This simplification can obscure the true structural drivers of health inequality, such as discrimination and unequal access to care. For groups with multiple marginalized identities—such as low-income, older women from ethnic minorities residing in remote areas—diagnostic outcomes may already exhibit systemic biases. Pursuing ever-more granular subgroup classifications to mitigate bias further exacerbates data scarcity and resource constraints, making it harder to develop reliable models for precisely those most in need of improved care (102).

Although AI has potential to improve global healthcare, addressing these ethical challenges is essential to ensure credible applications that respect human values and rights (113–115). AI and robotics can promote independence, monitor health and enhance social interactions in older adults (116). Human AI-enabled care systems have the potential enrich education, expand therapeutic options, and enhance clinical-patient relationships (117). Future research should focus on combining AI technologies with compassionate, human-centered care to improve patient experience and build trust, all while maintaining rigorous ethical oversight.

### 4.5 Establishing an ethical framework for fair AI

Despite AI’s demonstrated potential in managing frailty and coexisting conditions in older adults, its application must be governed by a robust ethical framework to ensure technological advancement does not come at the expense of fairness (72). In the face of profound challenges related to data privacy, interpretability limitations, complex algorithmic bias, and the global validation gap, a proactive and systematic governance approach is required. To address these issues, we propose a triple-ethical framework for fair AI, designed as a comprehensive structure for ethical oversight and responsible implementation.

#### 4.5.1 Intersectional data framework

AI systems must integrate diverse determinants of health beyond clinical and biomedical data, including socioeconomic status, cultural background, lifestyle, and environmental factors. Health inequalities among older patients are rarely attributable to a single factor, such as race or gender, but rather result from overlapping identities, including low income, minority ethnicity, and female gender. Consequently, AI data frameworks cannot treat populations as discrete subgroups; they must be capable of analyzing how these compounding structural inequalities collectively impact health outcomes for older adults.

#### 4.5.2 Explainability and auditability

“Black box” algorithms are unacceptable in high-stakes domains like older adults’ care. To ensure clinicians’ understanding and patients’ trust, AI recommendations (e.g., treatment adjustments) must be explainable. Clinicians need to comprehend the logic behind specific AI suggestions to critically evaluate them. In situations where AI systems—particularly agentic AI with autonomous learning capabilities—make decisions that affect patient care, it is essential to maintain a transparent, auditable decision pathway. This facilitates clinical and legal accountability when outcomes deviate from expectations.

#### 4.5.3 Ethical governance structures

Fairness cannot be an afterthought; it must be embedded within AI governance from the outset. We recommend a “transdisciplinary” governance model with the following elements.

(1) Proactive data management: Adopting a “privacy-first” principle in data handling. For example, use FL or differential privacy techniques to train AI models while safeguarding older patients’ sensitive data (e.g., genomic information, socioeconomic status).

(2) Mandatory human oversight: Ensure that AI serves as an augmentative tool for clinicians, not a replacement. Establish Clear processes for human intervention and veto in critical decision-making, especially at points involving ethical trade-offs.

(3) Routine fairness audits: Form independent ethics committees and transdisciplinary teams (including social scientists, ethicists, and patient representatives) to regularly audit AI algorithms. These audits should use intersectional fairness metrics to ensure that AI tools do not systematically disadvantage any sub-population of older populations (102).

### 4.6 Concrete protocols for ethical AI deployment

To operationalize ethical principles in clinical AI, we recommend enforcing three mandatory deployment protocols.

#### 4.6.1 Cross-group fairness stress testing

Prior to deployment, models must undergo performance testing across intersecting vulnerable populations (e.g., older adults in poverty). Clinical approval should be granted only when performance discrepancies remain below a predefined safety threshold (e.g., <5%) (118).

#### 4.6.2 Standardized clinical model cards

Each model must include a standardized disclosure specifying its intended use, known limitations, and an interpretable natural-language summary of the decision logic, enabling clinicians to clearly identify safe boundaries for application (119).

#### 4.6.3 Human-in-the-loop fail-safe mechanism

**Figure 3:**
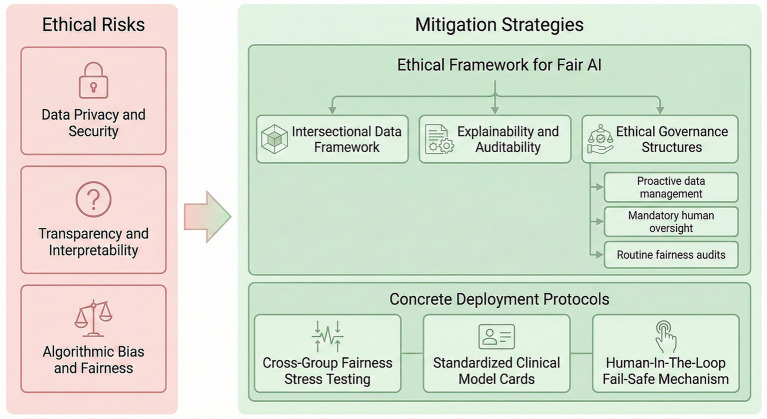
Mitigating ethical risks in AI. The figure outlines key ethical challenges on the left versus mitigation strategies on the right. The strategies encompass a triple-ethical framework—“Ethical Framework for Fair AI” for comprehensive oversight, supported by actionable “Concrete Deployment Protocols” such as cross-group fairness stress testing, standardized clinical model cards, and human-in-the-loop fail-safe mechanisms.

When risk indicators or recommendations exceed high-risk thresholds, the system must automatically lock decision outputs and require mandatory human review and reauthorization, while recording intervention data for traceability and safety auditing (Figure 3).

## 5 Future directions and conclusion

The future advancement of AI in managing multimorbidity among older adults should not rely solely on continuous algorithmic iteration. Instead, future progress must be guided by a systematic and transdisciplinary framework and fosters technological innovation while addressing ethical, organizational, and social challenges. Achieving this equilibrium requires structural reform at both organizational and governance levels. This section outlines several pathways toward realizing this vision (5).

### 5.1 AI new paradigm of transdisciplinary collaboration

The MDTs play a vital role in the management of complex geriatric cases, especially those multiple chronic conditions. AI technologies can substantially enhance the efficiency of MDT collaboration and the development of individualized treatment strategies (120). Despite their promise, AI-augmented MDTs still face challenges, particularly regarding ethical considerations and rigorous validation requirements (121). As Tan and Benos note, engineers and data scientists, often risk reducing complex human and societal issues to quantifiable parameters when addressing ethical concerns, thus stripping them of essential social dimensions. To avoid this reductionism, AI development should adopt a participatory and transdisciplinary approach from the outset—actively involving social scientists, ethicists, public health experts, and, crucially, patients and marginalized groups themselves. Only through such transdisciplinary collaboration can we ensure that technological innovation aligns with social values and healthcare priorities rather than unintentionally amplifying existing inequities (102, 122).

In practice, AI can integrate heterogeneous data from multiple hospital departments to generate comprehensive patient profiles that facilitate more efficient clinical decision-making (123). However, a professional and epistemic gap persists between data scientists and clinicians. Bridging this divide is essential: data scientists can help translate complex technical concepts for healthcare stakeholders, while clinicians provide domain expertise that grounds algorithmic insights in medical relevance (122). In radiology, collaboration between radiologists and data scientists is critical. Radiologists contribute anatomical and clinical expertise, whereas data scientists provide the computational and analytical tools necessary for model optimization (124). Through iterative interdisciplinary feedback loops, AI models can uncover novel diagnostic patterns or biomarkers, while clinicians refine algorithmic outputs and ensure clinical interpretability. Such bidirectional learning not only enhances model accuracy but also cultivates mutual trust between disciplines (122). Nevertheless, barriers to clinical adoption remain, emphasizing the need for ongoing efforts to improve AI interpretability and seamless integration into medical workflows (123). This form of interdisciplinary collaboration also serves as the organizational foundation for overcoming barriers to data interoperability. The alignment of technical standards—such as FHIR or OMOP—is not merely a technical task, but a socio-political coordination process (125). It requires clinicians, data scientists, hospital administrators, and policymakers to jointly establish shared data definitions and governance rules within a cross-disciplinary framework (126).

### 5.2 Next, steps in applications: multimodal data fusion and AI

AI has emerged as a transformative force for integrating multimodal biomedical data, particularly in the context of older adults with complex comorbidities. Recent research highlights the value of combining EHRs with medical imaging and omics data to improve clinical outcomes (127). This multimodal fusion enables a more comprehensive understanding of patient health, advancing both precision medicine and personalized care (43, 128).

Healthcare 5.0 framework has consistently demonstrated superior performance over unimodal systems in diverse healthcare applications (5, 129). Studies confirm that multimodal fusion models—especially those employing early fusion strategies—outperform single-modality approaches in disease diagnosis and prognosis (127). By integrating diverse data sources such as radiology, genomics, and EHRs, these models improve diagnostic precision and predictive robustness (123). AI techniques, particularly ML and DL, are well-suited to fusing such heterogeneous datasets (127). However, to achieve genuine social awareness and clinical inclusiveness, future multimodal AI must incorporate broader “Social Determinants of Health (SDOH)”—including social, environmental, behavioral, economic, and structural factors. Among older adults, comorbidities are profoundly shaped by lifestyle, living environments (e.g., air quality and food accessibility), social support networks, and systemic inequalities such as differential access to healthcare. Neglecting these factors risks producing biased models and exacerbating disparities. Hence, future AI-enabled multimodal frameworks must integrate these real-world determinants to build precision care systems that authentically represent patients’ lived experiences.

A fundamental bottleneck in applying AI to polypharmacy management among the older adults is the lack of standardized, interoperable healthcare data infrastructure (130, 131). Fragmented longitudinal health records—caused by incompatible EHR systems—hinder data continuity (130). Furthermore, multi-source datasets, such as clinical data, imaging (e.g., PACS), genomics, and wearable sensor data, are frequently isolated within disconnected “data silos.” The deeper issue is semantic interoperability—the consistent meaning of data across institutions. Variations in terminologies, ontologies, and coding systems obstruct accurate data integration (130, 131). These structural limitations constrain AI deployment by preventing large-scale, high-quality dataset construction and limiting generalizability across institutions, potentially reinforcing systemic bias (132). Therefore, before pursuing increasing complex AI models, the establishment of a universal interoperability framework—based on standards such as FHIR or OMOP—should be a prioritized as a foundation for sustainable AI-driven healthcare transformation (130, 133).

**Figure 4:**
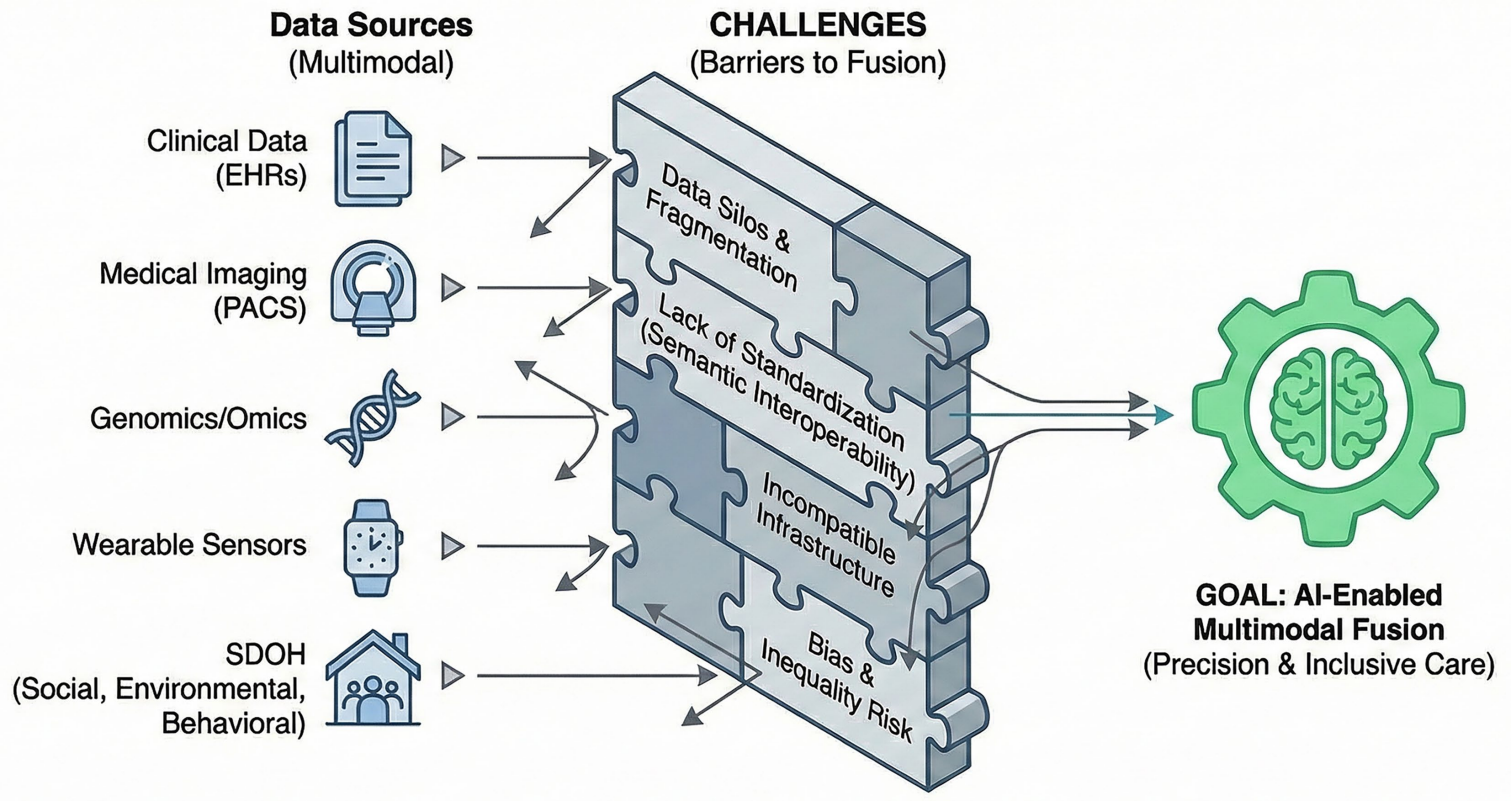
Barriers to integrating multimodal data for AI in multimorbidity. This figure illustrates fundamental infrastructural bottlenecks hindering the effective fusion of diverse data sources. EHRs, imaging, genomics, wearable sensors, and critical SDOH are often isolated within “data silos.” Structural limitations—such as fragmentation and lack of interoperability—that collectively block the path to truly integrated AI analysis and deployment.

Healthcare 5.0 framework integrates emerging technologies such as quantum computing, AGI, the IoT and 6G connectivity to enable hyper-personalized healthcare delivery (5, 129). By fusing multimodal data, AI facilitates a transition from data to wisdom—enabling predictive, preventive, personalized, and participatory medicine (111). This paradigm synthesizes genetic, lifestyle and environmental data through real-time data analytics to dynamically adjust care strategies (5). However, data preprocessing, model interpretability, and privacy protection remain key challenges to realizing the full potential of multimodal AI in healthcare (111, 128). As these technologies mature, they will reshape clinical research and delivery, particularly in areas such as personalized medicine, remote monitoring, and digital clinical trials (128) (Figure 4).

### 5.3 Policy and regulatory enablers

The AI holds tremendous potential to revolutionize healthcare diagnosis, treatment, and management (134). Yet, the absence of standardized regulatory frameworks continues to hinder safe and widespread implementation (135). Persistent concerns—including data privacy, ethical transparency, and the algorithmic accountability—remain key barriers (136). To address these issues, global health authorities must develop comprehensive and harmonized guidelines governing AI development, validation and deployment (135). In China, the integration of AI in older adults’ care services is promising, with 83% of older adults expressing willingness to adopt AI-driven tools. However, targeted policy support is necessary to ensure effective integration and trust-building (137). Globally, there is a growing recognition that existing regulatory systems often lag behind technological innovation. Thus, it is crucial that governance frameworks explicitly represent the needs and rights of older adults, ensuring inclusivity in AI-driven healthcare (137). Regulatory bodies such as the U. S. Food and Drug Administration (FDA) have begun publishing guidelines to improve AI transparency, interpretability, and validation, aiming to enhance clinical safety and oversight (138). Nonetheless, the success of AI-enabled geriatric care depends on aligning innovation with patient protection. Policymakers must simultaneously address data standardization, ethical oversight, and equitable access while safeguarding against bias and privacy breaches (139, 140).

Because AI depends on large-scale data, effective data-sharing frameworks are critical. The European Union’s General Data Protection Regulation (GDPR) exemplifies a balance between patient privacy and responsible data sharing (141). Such frameworks enhance AI model generalizability by enabling cross-institutional collaboration under robust privacy safeguards. However, striking a balance between innovation and protection remains difficult—overly restrictive policies can stifle progress and waste public investment (142, 143). Emerging solutions, such as transfer learning, synthetic data generation, and blockchain, show potential but remain underdeveloped for clinical implementation.

While AI-enabled care presents extraordinary opportunities for improving efficiency, personalization, and population health (144). It also raises ethical risks—including depersonalization, discrimination, dehumanization, and surveillance-based control (72, 145). To mitigate these risks, stakeholders across sectors—patients, clinicians, engineers, ethicists, and policymakers—must co-develop robust ethical frameworks emphasizing transparency, accountability, and patient-centered care (72, 146).

Ultimately, sustainable development of AI-enabled geriatric care requires balancing innovation with safety, fostering international cooperation on regulation, and promoting fair data sharing. By aligning technological advancement with ethical integrity and humanistic values, AI-enabled precision medicine can truly empower the future of healthcare for aging societies.

## 6 Discussion

Driven by population aging, declining birth rates, and mounting economic pressures, many societies face a dual crisis of increasing healthcare demand and shrinking labor supply. Traditional, human-intensive models of care are becoming unsustainable.

We underscore the promising role of AI-enabled precision medicine in improving the management of multimorbidity in older adults. By integrating ML, NLP, CV, and big data analytics, agentic AI is driving a shift from fragmented, disease-centered care toward more proactive, personalized, and holistic healthcare models. The core driver of this transformation is agentic AI, which functions as a crucial bridge between the macro vision of Healthcare 5.0 and real-world clinical implementation. By autonomously mitigating fragmented care and reducing administrative burdens, agentic AI enables older adults care to become both sustainable and adaptive, even amid persistent workforce shortages. These innovations support early risk prediction, tailored treatment planning, enhanced self-management, and more efficient use of healthcare resources.

Despite encouraging progress, significant challenges remain. Hyper-personalized medicine continues to face major ethical challenges: data privacy, health equity, system reliability, and the reconfiguration of doctor-patient relationships. Infrastructure limitations and a lack of standardized frameworks also hinder large-scale implementation. Fragmented data systems, inconsistent data quality, and poor interoperability complicate multimodal data integration and cross-institutional collaboration. Furthermore, limited AI literacy among healthcare professionals creates additional barriers to adoption. The “black-box” nature of AI models makes it difficult for clinicians to interpret results and trust AI-driven recommendations.

Achieving the full potential of AI in older adults multimorbidity management requires operationalizing the Healthcare 5.0 principles at both technological and policy levels. Future research should move beyond algorithmic performance to develop sustainable, human-centered ecosystems aligned with this vision. AI-enabled precision medicine is not just a technological innovation—it represents a fundamental shift toward more compassionate, efficient, and inclusive healthcare for older populations. At the policy level, adaptive governance is needed to ensure ethical use, protect patient privacy, and promote equitable access to AI-enabled care. Aligning innovation with ethical principles and patient-centered values will be key to ensuring that these technologies benefit all, especially the most vulnerable members of society.
